# Bilirubin: translational perspectives

**DOI:** 10.1038/s41390-025-04432-z

**Published:** 2025-09-19

**Authors:** Libor Vítek

**Affiliations:** 4th Department of Internal Medicine and Institute of Medical Biochemistry and Laboratory Diagnostics, 1st Faculty of Medicine, Charles University and General University Hospital in Prague, Prague, Czech Republic

## Abstract

**Abstract:**

Bilirubin, an old tetrapyrrolic compound that had occurred on Earth early on, is the final product of the catabolic heme pathway in the intravascular bed. Data from recent decades revealed its enormous bioactivity in a human body, including antioxidant, anti-inflammatory, immunosuppressive, antiproliferative, and even cell signaling activities that translate into beneficial effects of mildly elevated serum bilirubin concentrations resulting in prevention or amelioration of progression of many diseases of civilization. Furthermore, recent advances in bilirubin research have changed our understanding of bilirubin metabolism in the neonatal period, with discoveries of bilirubin reductase of bacterial origin in the intestinal lumen with direct pathophysiological and clinical implications. Similarly, our knowledge of the pathophysiology of neonatal jaundice phototherapy has improved substantially, although we are still at the beginning of the path to understand all the pathophysiological aspects and reveal related clinical implications.

**Bullet points:**

- Recent advances in our understanding of bilirubin metabolism with clear clinical implications, as well as other, so far putative, translational impacts.
- Demonstration of the beneficial biological potential of bilirubin, its evolutionary and ontogenetic functions, its possible role in chronobiology, and its correlation with increased fitness in elite athletes (a sort of gain of function).
- Discussion on the protective role of physiological neonatal jaundice.
- Inspiration for further basic and clinical research in specific fields of bilirubin metabolism.

## Versatile biological functions of bilirubin: from phylogenesis to ontogenesis, from biological activities to clinical impacts

Since the major discoveries of heme structure, bilirubin, and biliverdin synthesis made by a genius German chemist Hans Fischer (1930 Nobel Prize in Chemistry laureate for his contributions to the field of porphyrin chemistry), bilirubin has been thought for decades to be a waste, potentially dangerous product of the heme catabolic pathway.

However, based on intensive research in bilirubin metabolism over the last 40 years, bilirubin now appears to be a versatile bioactive molecule with multiple functions (Table 1).

**Table 1:** Versatile biological functions of bilirubin.

Biological activity of bilirubin	Clinical impact	References
Oxidative stress defense	Prevention of diseases associated with increased oxidative stress	^1^
Immunosuppressive activities	Prevention of autoimmune diseases and diseases associated with low grade chronic inflammation	^2^
Antimicrobial effects	Protection from infections	^29–34^
Cell signaling activities	Protection from metabolic disorders	^6^
Anticancer effects	Protection from most of cancer diseases	^25–28^
Exerkine functions	Improving physical fitness	^10,11^
Role in chronobiology	Regulation of the human circadian clock Possible link to seasonal affective disorders	^12–16^
Role in ontogenesis	Improvement of oocyte maturation	^19,20^

Bilirubin belongs to the phylogenetically old superfamily of heme-derived tetrapyrrolic compounds. It is a product of the degradation of heme in the intravascular bed and plays an enormous role in the regulation of various biological processes in a human body.

Bilirubin has a role in oxidative stress defense,^1^ being the most potent endogenous antioxidant substance. This property, together with its general immunosuppressive activities,^2^ translates into suppression of pro-inflammatory status, and low-grade chronic inflammation that is a common pathophysiological denominator of many diseases of civilization.^3^ In fact, this has been repeatedly proven in many recent clinical studies and summarized in recent review articles.^4,5^

Very recently, bilirubin has begun to be viewed as a molecule with potent signaling activities^6^ acting as a real hormonal substance^7^; revealing further beneficial pathways that contribute to the prevention or amelioration of many diseases of civilization. This has been convincingly proven in subjects with Gilbert´s syndrome (benign hyperbilirubinemia),^8^ who are protected against the development of atherosclerotic,^9^ as well as other diseases.^4,5^ Bilirubin can even act as an exerkine, improving physical fitness, as reported in our recent study in elite athletes^10^ and in a recent review paper.^11^ In this context, it is tempting to speculate that increased fitness associated with mild hyperbilirubinemia may represent a sort of gain of function with possible evolutionary advantages for subjects with Gilbert´s syndrome, although so far no data on this hypothesis have been reported.

However, bilirubin appears to have other putative biological activities that await to be explored in deeper detail. Similar to other related tetrapyrrolic pigments that occur in nature, bilirubin may have an important role in chronobiology, acting as a regulator of the human circadian clock. This is based on older observations that popliteal illumination leads to a shift in circadian rhythms, with changes in bilirubin.^12–14^ In addition, bilirubin concentrations change during night. Interestingly, exposure to light during night increases serum bilirubin concentrations suggesting the link to seasonal affective disorders.^15,16^

Interestingly, bilirubin (and its precursor biliverdin) may play a role in ontogenesis, as suggested by studies on sea lampreys.^17^ The role of biliverdin in ontogenesis has also been reported in lower organisms such as *Xenopus laevis*, in which biliverdin binds to highly conserved yolk proteins vitellogenin and lipovitellin that are important for embryogenesis.^18^ Although detailed data in humans are lacking, it is also possible that biliverdin and bilirubin-IXα may play a developmental role in the human fetus, since these are the predominant bile pigments in fetal life. In fact, bilirubin was demonstrated to improve mouse oocyte maturation in vitro,^19,20^ suggesting not only its role in oogenesis but also providing an innovative strategy to enhance oocyte in vitro maturation.

Bile pigments are evolutionary very conserved structures; they appeared in the simplest organisms such as algae and cyanobacteria.^21^ In fact, the cyanobacterium *Synechocystis sp*., fully equipped with heme oxygenase and biliverdin reductase, appeared on Earth as early as 3.5 billion years ago.^22^ It is certainly interesting that bilirubin-related tetrapyrrolic compounds present in the blue-green alga *Spirulina platensis* were proven to have apparent anticancer effects,^23^ and the same was demonstrated for chlorophyll, another tetrapyrrolic pigment so prevalent on Earth.^24^ Indeed, bilirubin per se is believed to drive protection against various cancer diseases,^25–27^ as summarized in a recent review paper.^28^

Apart from their immune system modulating activities, bile pigments and their precursors appear to have direct antimicrobial effects. Interestingly, this has been demonstrated even in lower animals, such as birds, in which the deposition of porphyrin-like pigments in the egg shells provides protection against Gram-positive bacteria,^29^ as well as the feared coronaviral poultry infections.^30^ However, even more importantly, bilirubin has been shown to have antibacterial activities related to human infections. As early as 1937, Najib-Farah described ¨the defensive role of bilirubinaemia in *Pneumococcal* infection¨.^31^ Much later, Hansen et al. reported inhibitory effects of bilirubin on the growth of group B *Streptococci*, the most common bacteria involved in early-onset neonatal sepsis,^32^ suggesting that physiological jaundice may have an evolutionary role in protecting against early-onset neonatal sepsis. The whole context of antimicrobial effects of bilirubin is even wider, as evidenced by the very recent observation of antimalarial effects of bilirubin,^33,34^ which can again have even evolutionary impact.

## Bilirubin reduction in the gut lumen

Very recently, the missing puzzle has been discovered in the entire catabolic pathway of heme, that is, reduction of bilirubin to urobilinoids in the intestinal lumen.^35^ Using a combination of biochemical analyses and comparative genomics, Hall et al. identified a novel enzyme, BilR, which can reduce bilirubin to urobilinogen. They delineated the BilR sequences from other members of the so-called Old Yellow Enzyme family by identifying key residues at the active site that were critical for the reduction of bilirubin and found that BilR is predominantly encoded by *Firmicutes* in the gut microbiome.^35^ This discovery appears to be of paramount importance since the absence of bilirubin reduction in the intestinal lumen was shown to be directly implemented in the pathogenesis of neonatal jaundice,^36^ and intestinal metabolism of bilirubin by the gut microbiota was demonstrated to be directly linked to serum bilirubin concentrations.^37^ Therefore, it is not surprising, that modulation of the gut microbiota is at the center of efforts to ameliorate the severity of neonatal jaundice. In fact, the use of probiotics as a tool to colonize the neonatal gut with favorable bacteria appears to be a viable approach with great therapeutic potential,^38,39^ particularly when taking into account that no specific probiotic bacteria that reduce bilirubin have been developed and used in clinical studies so far.

## Neonatal jaundice and phototherapy

It is now well recognized that physiological neonatal jaundice is a functional state that provides systemic protection against the oxidative challenge due to birth, increasing the risk of various complications mediated by postnatal oxidative stress (for a review, see ref. ^40^). In fact, neonatal hyperbilirubinemia increases,^41^ while phototherapy reduces blood antioxidant capacity, inducing significant oxidative stress and pro-inflammatory status with increased production of inflammatory cytokines.^42^ Hence, these facts should be taken into account and perhaps become a basis for future improvement of the criteria for starting phototherapy.

On the other hand, research on neonatal jaundice phototherapy has recently been advanced, confirming the efficacy and superiority of blue-green phototherapy over blue light, both in basic research^43^ as well as in clinical trials.^44,45^ However, phototherapy for neonatal hyperbilirubinemia appears to be associated with certain risks of developing cancer and other diseases, and also higher mortality in extremely low birthweight neonates.^46^ Several putative reasons have been suggested, including the contribution of bilirubin photooxidation products, whose role despite certain progress in the research revealing their neuroinflammatory, oxidative stress-evoking and metabolic effects.^47–50^ It is clear, however, that further research is needed to fully uncover all unknown facts and mechanisms beyond phototherapy of neonatal jaundice. In this sense, a great analytical and pathophysiological tool appears on the horizon with successful and efficient synthesis of all major bilirubin oxidation products^51^ with the promise of using this synthetic platform for the development of stable isotope-labeled bile pigments. This, without question, would advance our attempts to understand the kinetics and biological behavior of all products generated by neonatal jaundice.

In conclusion, bilirubin and other tetrapyrrolic compounds that occur in nature exert multiple biological activities, modulating various functions in the human body. From a wider perspective, this field will certainly grow rapidly in the near future, and we will probably call bilirubin hepatokine, neurokine, metabolokine, or even exerkine. Furthermore, recent advances in our knowledge of bilirubin metabolism during neonatal age will certainly translate into improved clinical care for jaundiced newborns.

## Data Availability

All data discussed are included in the manuscript.
